# PRNU-Based Video Source Attribution: Which Frames Are You Using?

**DOI:** 10.3390/jimaging8030057

**Published:** 2022-02-25

**Authors:** Pasquale Ferrara, Massimo Iuliani, Alessandro Piva

**Affiliations:** 1 European Commission—DG Joint Research Centre, 21027 Ispra, Italy; pasquale.ferrara@ec.europa.eu; 2 Department of Information Engineering, University of Florence, Via di S. Marta 3, 50139 Florence, Italy; massimo.iuliani@unifi.it; 3 FORLAB, Multimedia Forensics Laboratory, PIN Scrl, Piazza G. Ciardi 25, 59100 Prato, Italy; 4 National Inter-University Consortium for Telecommunications (CNIT), Viale Usberti, 43124 Parma, Italy

**Keywords:** video forensics, video source attribution, sensor noise, digital stabilization

## Abstract

Photo Response Non-Uniformity (PRNU) is reputed the most successful trace to identify the source of a digital video. However, its effectiveness is mainly limited by compression and the effect of recently introduced electronic image stabilization on several devices. In the last decade, several approaches were proposed to overcome both these issues, mainly by selecting those video frames which are considered more informative. However, the two problems were always treated separately, and the combined effect of compression and digital stabilization was never considered. This separated analysis makes it hard to understand if achieved conclusions still stand for digitally stabilized videos and if those choices represent a general optimum strategy to perform video source attribution. In this paper, we explore whether an optimum strategy exists in selecting frames based on their type and their positions within the groups of pictures. We, therefore, systematically analyze the PRNU contribute provided by all frames belonging to either digitally stabilized or not stabilized videos. Results on the VISION dataset come up with some insights into optimizing video source attribution in different use cases.

## 1. Introduction

Video source attribution is commonly addressed by extracting the traces left into the content by the Photo Response Non-Uniformity (PRNU), originated by manufacturing processes in the form of slight imperfections in light response of pixels, and by comparing them to a reference trace characterizing the device. This methodology, firstly applied for image source attribution [1,2], provided outstanding results in several contexts, even when the source or questioned device is not available [3,4], as well as in large-scale scenarios [5], or when an image is exchanged through social media platforms [6,7].

Few years later, the same technology was extended to digital videos [8,9]. The first video source attribution schemes consisted of estimating and comparing two PRNU from a reference and a query video, respectively. However, commonly-used devices, such as smartphones, usually provide strongly compressed videos with limited resolutions. This fact makes PRNU estimation less reliable, even in the presence of long video recordings. The main reason behind this is that most frames, being strongly compressed, slightly contribute to the estimate of the noise pattern.

Several solutions have been proposed to cope with video compression: in [10], the authors proposed a confidence weighting scheme to manage videos where high-frequency contents (e.g., edges) persist at a given image location. In [11] proposed a block-based approach to build a more reliable PRNU pattern. In [12] the authors noticed that intra-coded (I) frames are more reliable than predicted (P) frames, and they assign different weights to the two kinds of frames to achieve higher accuracy with fewer frames. The efficiency of a method based only on I frame is also shown in [13], where the authors also highlight that recent cameras can automatically rotate a video 180 degrees while recording with rolling 180 degrees and thus introducing another complexity in the attribution process. In [14] the authors propose an approach that exploits only those frame blocks with at least one non-null DCT-AC coefficient. In the latest years, Altinisik et al. [15] proposed to mitigate the disruptive effects of video compression on PRNU estimation by intervening in the decoding process. The aim was to eliminate any filtering procedure applied at the decoder, in order to reduce the blockiness. In this work, they also noticed that the prediction error that will be subject to quantization could be of lesser strength for P and Bidirectional predicted (B) frames. Therefore, P and B frames can contain relevant PRNU information at the same compression level of I frames.

However, all the mentioned works do not consider devices equipped with electronic image stabilization (EIS), which aimes at compensating for camera shake. The technical specifics of this compensation are generally based on proprietary software, and their implementation details usually vary among brands. In few cases, some differences among the existing EIS implementations have been studied in controlled environments and pointed out [16]. However, from a technical point of view, we can consider all these techniques as image registration approaches [17] mainly comparable to linear transformations, such as rigid or affine. The parameters of such transformations can be estimated via brute-force or through some registration techniques [18]. In [19], a computer vision perspective is proposed, by exploiting deep learning based registration techniques, to determine scaling and rotation transformations.

Note that when EIS is applied, PRNU is transformed accordingly, being solid with respect to the transformation, and this issue has to be considered during PRNU comparison. Starting from this consideration, in [20], the authors intuitively assume that I frames are still the best to be used with stabilized videos. Furthermore, Taspinar et al. emulate in-camera stabilization with third-party software, thus making their results still far from actual cases. Ferrara et al. analyze modern smartphones from VISION Dataset [21] and propose to compute the fingerprint from the most representative frames that, once accumulated, maximize the peak-to-correlation energy (PCE) [22]. The main drawback is the growth of false alarm rate due to the maximization step. In [18], the authors notice that the first frame of a video sequence is generally not affected by EIS, thus making it effective by itself to compute a reliable fingerprint estimate. This fact also suggests that the first group of pictures (GOP) can be less affected by digital stabilization, thus providing important PRNU information. Nevertheless, the authors consider I frames only, based on the results in the previous works. Eventually, Altinisik et al. [23], focusing on I frames, introduce a source camera verification method for strongly stabilized videos, by assuming more degrees of freedom and taking into account the spatially variant nature of EIS. In the VISION and other datasets, they correctly verify the source of 23–30% of the strongly stabilized videos available. The main drawbacks of the method are its high computational cost for the blind inversion step and the rise of false PRNU matching due to content similarity among frames. Overall, it is still not clear whether there is a general and optimum strategy to select frames within a video sequence in the case of digitally stabilized videos. In this paper, we systematically analyze the PCE on several video sequences, including digitally stabilized videos, to determine whether: (i) I frames always produce a better fingerprint estimate than P frames; (ii) the quality of a fingerprint estimated from P frames depends on the frame position within the GOP; (iii) the first GOP should be preferred for a better fingerprint estimate.

The paper is organized as follows: Section 2 recalls technical fundamentals of video compression; Section 3 provides an introduction of PRNU and describes the methodology that we used to conduct our study. Section 4 reports the performed experiments, while Section 5 summarizes the main findings and insights of our analysis. Finally, Section 6 draws the conclusions and highlights the open issues.

## 2. Basics of Video Compression

Before going into the details of source camera attribution based on PRNU, we briefly recall some basics of video compression. Any digital video is essentially a temporal sequence of pictures (i.e., frames) acquired at a given frame rate. Such a data stream can be compressed to save storage or transmission bandwidth by exploiting spatial and temporal redundancies and, at the same time, by considering the characteristics of the Human Vision System (HVS) in order to minimize visual degradation. To achieve this goal, the most common video compression standards such as Advanced Video Coding (also known as MPEG-4 AVC or H.264) [24,25], or the more recent H.265 [26], employ block-oriented and motion-compensated encoding approaches. Briefly, these coding schemes divide frames into two different types: intra-coded frames, also referred as I frames, and predictive-coded frames, which can be divided in sub-types such as predicted (P) and bidirectional predicted (B) frames. During the encoding process, frames are grouped in GOPs (group of pictures). Every GOP always begins with an I frame and then presents a certain number of predictive frames. The number of frames composing a group of pictures is called GOP size, which can be constant or variable depending on the specific implementation. In modern smartphones, GOPs generally have a fixed number of frames (e.g., 30) and do not include B frames, thus making available a fixed percentage of I and P frames, respectively.

When a frame is compressed, the encoder divides it into macroblocks (MBs), and encodes each MB individually: MBs belonging to I frames are always encoded without referring to other frames, by means of a DCT quantization strategy. In this sense, I frames are encoded in a similar way as JPEG images. The main difference resides in the MB size, bigger than the typical 8×8 pixels JPEG block, and in the quantization coefficients applied to the DCT. Compared to a JPEG image whose quality factor is 100%, quantization coefficients of an intra-coded MB are larger, so that most of the high frequency components are filtered out.

On the contrary, MBs belonging to predictive-coded frames may be encoded referring to previous frames (this is the only possibility in P frames) or even referring to the following frames (allowed in B frames). Besides predicted MBs, the encoder embeds the motion vectors associated to each MB. In some configurations, the encoder has also the possibility to skip a MB in a predictive-coded frame, if this MB can be directly copied from a previous frame (e.g., in presence of a static content). Finally the numeric stream is further compressed by means of lossless compression.

For a more detailed explanation, reader might refer to [24,25,26].

## 3. Prnu Estimation

When dealing with digitally stabilized videos, it is convenient to estimate the camera fingerprint from still images because they are not affected by EIS [27].

**Figure 1:**
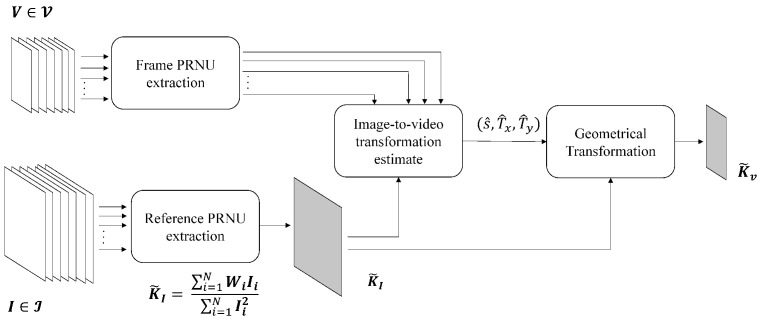
Pipeline describing the generation of the camera PRNU. The workflow is repeated for each device. The sets of images I and the video V are assumed to be acquired by the same device.

In the proposed analysis, we thus exploit still images to generate fingerprints of all available devices. The workflow for generating camera fingerprints is shown in Figure 1. Note that, when testing a query video, it is necessary to properly downscale and to crop the image fingerprint that is generally acquired by using a larger portion of the camera sensor, while when generating a video sequence, the full-frame sensor is generally downsized to reduce the amount of processed data (e.g., through pixel binning [28]). The scaling and cropping parameters of each device are estimated according to the methodology first introduced in [27], and then refined in [18,22].

Given a set I of *N* images $I_{1},\ldots ,I_{N}$, the camera fingerprint is estimated as follow: a denoising filter [1,29] is applied to the images to obtain the noise residuals $W_{1},\ldots ,W_{N}$; the reference camera fingerprint estimate ${\tilde{K}}_{I}$ is derived by the maximum likelihood estimator [30]:

(1) $${\tilde{K}}_{I}=\frac{\sum_{i=1}^{N}W_{i}I_{i}}{\sum_{i=1}^{N}I_{i}^{2}}$$

where the subscript I stands for a PRNU at the same resolution as the images. Two further processing are applied to ${\tilde{K}}_{I}$ to remove demosaicing traces, JPEG blocking, and other non-unique artifacts [30].

In parallel, the same denoising filters and processing are used to extract the noise patterns from video frames originated by the same camera as the set of images I. The noise patterns are processed individually in order to avoid the combination of geometrically misaligned frames when EIS is activated. For each frame, we estimated the parameters of scaling $\hat{s}$ and cropping $\left({\hat{T}}_{x},{\hat{T}}_{y}\right)$. The estimation is performed by means of exhaustive search over the parameters $\left(\hat{s},{\hat{T}}_{x},{\hat{T}}_{y}\right)$ that maximize the cross-correlation between the frame noise pattern and $K_{I}$. Once estimated at the level of single frame, the parameters are aggregated by calculating the most frequently estimated values, as suggested in [22]. Finally, we applied the geometrical transformation $T_{\hat{s},{\hat{T}}_{x},{\hat{T}}_{y}}$ to ${\tilde{K}}_{I}$ in order to obtain the camera fingerprint for video ${\tilde{K}}_{V}$.

**Figure 2:**
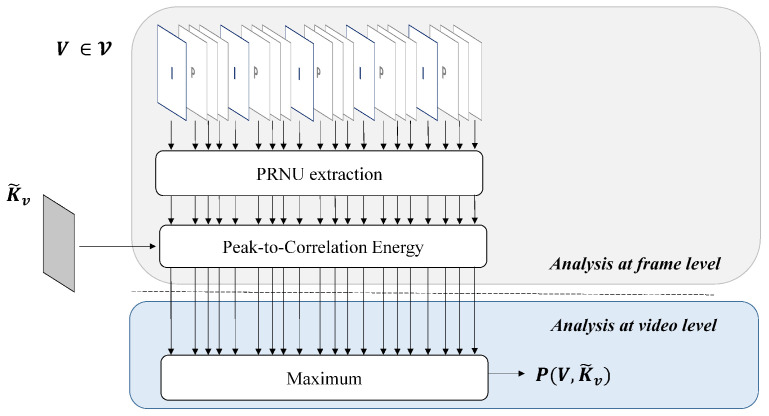
Pipeline describing the testing process between a query video V and the camera PRNU.

The testing procedure is described in Figure 2. Given a query video frame V, belonging to the video V, and a reference camera fingerprint ${\tilde{K}}_{V}$, the source attribution test is defined as follow: given W, the noise residual extracted from V, the two-dimensional normalized cross-correlation $\rho (s_{1},s_{2};W,{\tilde{K}}_{V})$ is computed for any plausible shift $\left(s_{1},s_{2}\right)$; then the PCE ratio [5] is derived as

(2) $$PCE\left(V,{\tilde{K}}_{V}\right)=\frac{\rho {\left(s_{peak}\right)}^{2}}{\frac{1}{mn-|N|}\sum_{s\notin N}\rho {\left(s\right)}^{2}},$$

where N is a small set of peak neighbours and (m,n) is the image pixel resolution, and $s_{peak}$ is the shift value that maximizes the correlation $\rho (s_{1},s_{2})$. In case of stabilized videos, the search space of the shift peak can be reasonably considered a square centered in (0,0), representing the plausible shift introduced by the EIS.

In general, a query video V comprises hundreds of frames $V_{1},\ldots ,V_{N}$, thus allowing to compute Equation (3) for all available frames. In the end, given *N* frames, we compute the test statistic

(3) $$P\left(V,{\tilde{K}}_{V}\right)=\max_{j=1,\ldots ,N}PCE\left(V_{j},{\tilde{K}}_{V}\right).$$

For a given threshold τ, we decide that the fingerprint ${\tilde{K}}_{V}$ is found within V, i.e., the video belongs to the reference camera, if P>τ.

For the sake of completeness we report the pseudo-codes of the algorithms for estimating the video fingerprint (Algorithm 1) and testing the video frames respectively (Algorithm 2).
**Algorithm 1** Fingerprint Generation.1:*input data:* for each device, $V_{1},\ldots ,V_{N}$, I frames, $I_{1},\ldots ,I_{m}$ still images;2:                   neighbourhood size N, PCE_threshold τ;3:                   minimum scale $s_{m}$, maximum scale $s_{M}$, scale increment $\delta_{s}$;4:*result:* Fingerprint of the inquired camera;5: 6:**for**1≤i≤m**do**7:    extract PRNU $W_{i}$ from $I_{i}$8:**end for**9: 10:apply Equation (1) among $I_{1},\ldots ,I_{m}$ and get ${\tilde{K}}_{I}$11: 12:**for** 1≤j≤n**do**13:    extract PRNU $W_{j}$ from $V_{j}$;14:    inizialize $T_{x}$, $T_{y}=0$;15:    **for** $s=s_{m}$; $s\leq s_{M}$ **do**16:          $s=s+\delta_{s}$;17:          compute $s(W_{j})$, scaled version of $W_{j}$ by *s*;18:          ${curPCE}_{j}=PCE_{j}\left(s\left(W_{j}\right),{\tilde{K}}_{I}\right)$19:    **end for**20:    **if** max(curPCE) >τ **then**21:          save ${\tilde{s}}_{j},{\tilde{T_{x}}}_{j},{\tilde{T_{y}}}_{j}$ corresponding to achieved max(curPCE)22:    **end if**23:    $s=mode(\tilde{s})$; $T_{x}=mode\left({\tilde{T}}_{x}\right)$, $T_{y}=mode\left(\tilde{T_{y}}\right)$24:**end for**25: 26:obtain $K_{V}$ by scaling and cropping $K_{I}$ by *s*, $T_{x}$, $T_{y}$
**Algorithm 2** Frame Testing.*input data:* video fingerprint reference $K_{V}$, for each device,2:$V_{1},\ldots ,V_{n}$ belonging to video V*result:* PCE between $K_{V}$ and each frame4: **for**1<l<n**do**6:    extract PRNU $W_{l}$ from $V_{l}$;     compute PCE between $K_{V}$ and $V_{l}$8:**end for**

## 4. Experiments

The experiments are performed on a computer with an Intel64 Family 6 Model 158 Stepping 9 GenuineIntel @ 3.601 GHz CPU and 32 GB RAM. The code for PRNU extraction and matching is developed in MATLAB.

Tests were performed on the VISION dataset [21], including images and videos from 34 devices (We excluded device D13 from the original dataset given the low resolution of the images and videos) belonging to 11 different brands: Apple, Asus, Huawei, Lenovo, LG Electronics, Microsoft, OnePlus, Samsung, Sony, Wiko, and Xiaomi. For each device, we produced a reference PRNU pattern by using the approach defined in [22]. For query data, we used the videos categorized as *indoor* and *outdoor* according to VISION names convention.

### 4.1. Experimental Setup

We systematically analyzed the PRNU contribution of each frame type. To do so, we extracted the PRNU from all video frames, and we determined the frame types by using *ffprobe*, a tool provided by FFmpeg (https://ffmpeg.org/ accessed on 12 February 2022). To be independent of the resolution, we computed the PCE between the reference and the frame PRNU by cropping a central patch of 480×480 pixels. The search space of the correlation peak within the correlation matrix was set at 30×30 pixels and centered around the expected peak position (ideally at (0,0) lag). Such a large search space allows to deal with possible horizontal and vertical translations of the query PRNUs due to EIS. Possible rotation and scaling factors are excluded since they do not severely affect the performance and they allow to keep the complexity reasonable [18].

Afterward, we performed a distinctive analysis in function of:

- whether the frame is an intra or a predicted coded one;
- whether the video is acquired in presence of EIS or not;
- whether the frame belongs or not to the first GOP.

The last distinction was made to reach a deeper understanding of some devices where EIS eventually is not applied to the first (intra-coded) video frame [18].

### 4.2. Evaluation at Frame Level

**Figure 3:**
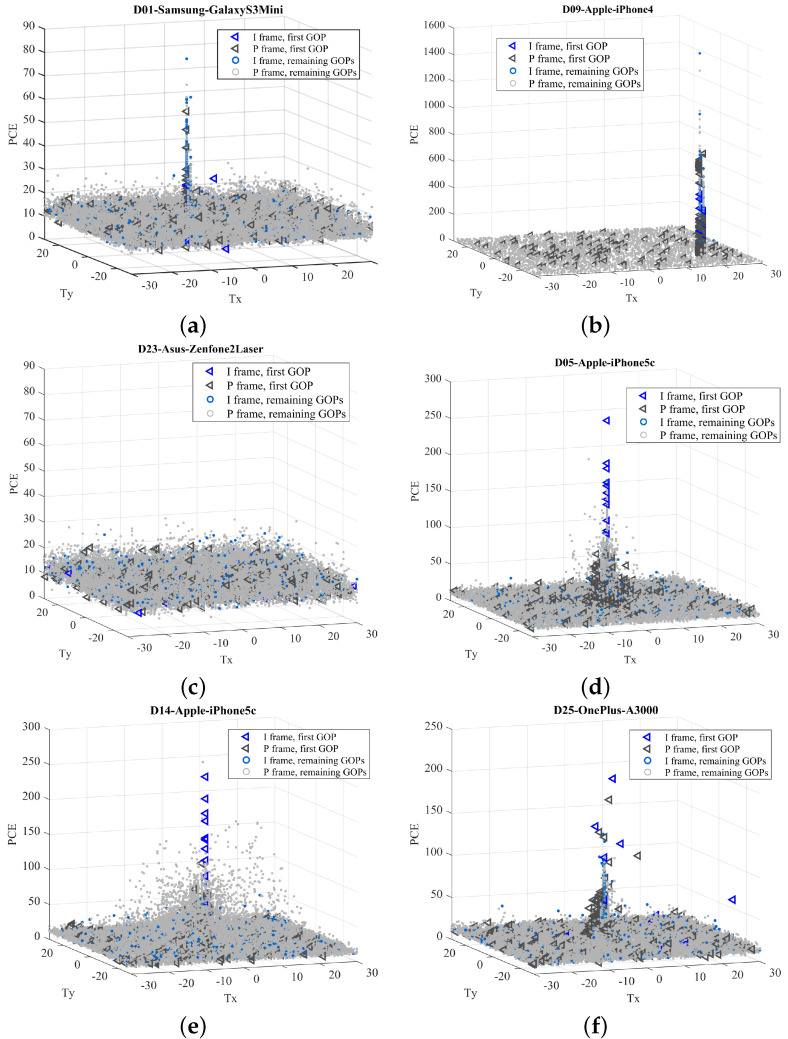
3D scatter plots of PCE in function of correlation peak position of six most representative devices. PCE is evaluated for each frame of all videos available for a given device. Samsung (**a**), Apple (**b**,**d**,**e**), Asus (**c**) and OnePlus (**f**) devices are shown. Non-stabilized (**a**–**c**), as well as stabilized (**d**–**f**) videos are presented. We also report a case of missed detection (**c**) at frame level.

First, we evaluated the PCE distributions grouped by devices. Results are shown in Figure 3: for the sake of space, we reported the most meaningful examples that summarize all the observed behaviors. In each subfigure, we plotted the PCE value in function of the correlation peak position $\left(T_{x},T_{y}\right)$. Each detected peak comes from the comparison between a frame and the reference PRNU coming from the same device. Different colors are used for I frames (in blue) and P frames (in gray), as well as distinct markers are adopted for frames belonging to the first GOP (triangle) and following GOPs (circle), respectively. The overall finding is that the achievable correlation energy strongly varies among devices, based on several factors: (i) the basic amount of sensor noise (that may vary among brands); (ii) the video acquisition settings and coding parameters (e.g., quantization tables) of the camera software; (iii) the estimation uncertainty suffered by the alignment compensation between the image and video PRNU. Although it is unlikely to predict the combined effects of these factors for each device, we try to provide some justification about the main behaviours (reported in Figure 3). In videos acquired from the same (or similar) device models, such as the iPhone 4 and 5c in (b), (d), and (e), the chances are that non-stabilized frames achieve higher PCE values than stabilized frames. This first conclusion is reached by considering that EIS introduces rounding errors among the relative positions of the acquired video frames. Then, the estimation of EIS parameters introduces a further uncertainty, resulting in a lower PCE value. Unfortunately, this assessment is hard to be done for (f) since we do not have any non-stabilized video of the same or similar models. Similarly, video frames in (a), (b), and (c), all non-stabilized, achieve very different PCE: devices in (a) and (c) achieve lower PCE ranges compared to (b). The small dynamics of D01 values can be explained by the effect of a strong compression. For device D23, it is likely that the reference PRNU is not extracted correctly. It worth also noticing that, for device D09 (not adopting EIS), we observe a significant offset between the reference PRNU for that device and the PRNU extracted from video frames. In this case, a small neighborhood (5×5 or 10×10) of search could lead to a missed detection.

Globally, in non-stabilized videos, frames tend to match in a position very close to the same PCE location (see Figure 3a,b), except for device D23. It is worth noting that in all cases there is no a significant ’preference’, neither for selecting I frames with respect to P frames, nor for using frames from the first GOP with respect to the ones belonging to the following GOPs.

The situation rather changes for stabilized recordings (Figure 3d–f). Commonly, PCE values tend to spread around a central position, following the fact that EIS compensates horizontal and vertical relative shifts between subsequent frames so that the PRNU of each frame is misaligned to each other. In most cases, the first frame contains discriminative information for the source attribution. However, while in some devices, the first video frame is not affected by stabilization (e.g., D05 and D14), in other cases, a motion compensation must be taken into account to avoid a missed detection (e.g., D25). In several devices, properly compensated P frames can also contribute to the PRNU information (e.g., D05, D14, D25). However, it is worth noticing that the PRNU contribution of each frame type (I and P) may strongly vary among devices. Overall, in Table 1 we report, the percentage of I and P frames providing a relevant PCE (τ>50) for each device. Devices equipped with EIS are highlighted in bold. In the last two columns, we also report the corresponding standard deviation of the estimated peak shifts. For instance, the shift alignment required to match the I frames of the iPhone 4s has a standard deviation of 6.16 and 9.87 pixels on the horizontal and vertical axes respectively. In some cases, we are not able to estimate them since we could not find any matching frames (e.g., P9 Lite). PRNU information can be found more easily on Apple devices and some fewer spread models (e.g., Lumia 640 LTE, Ridge 4G). Conversely, in Samsung and Huawei devices, the contribution of a single frame is rather enough to correctly identify the source, especially for intra-coded pictures.

### 4.3. Performance at Video Level

**Figure 4:**
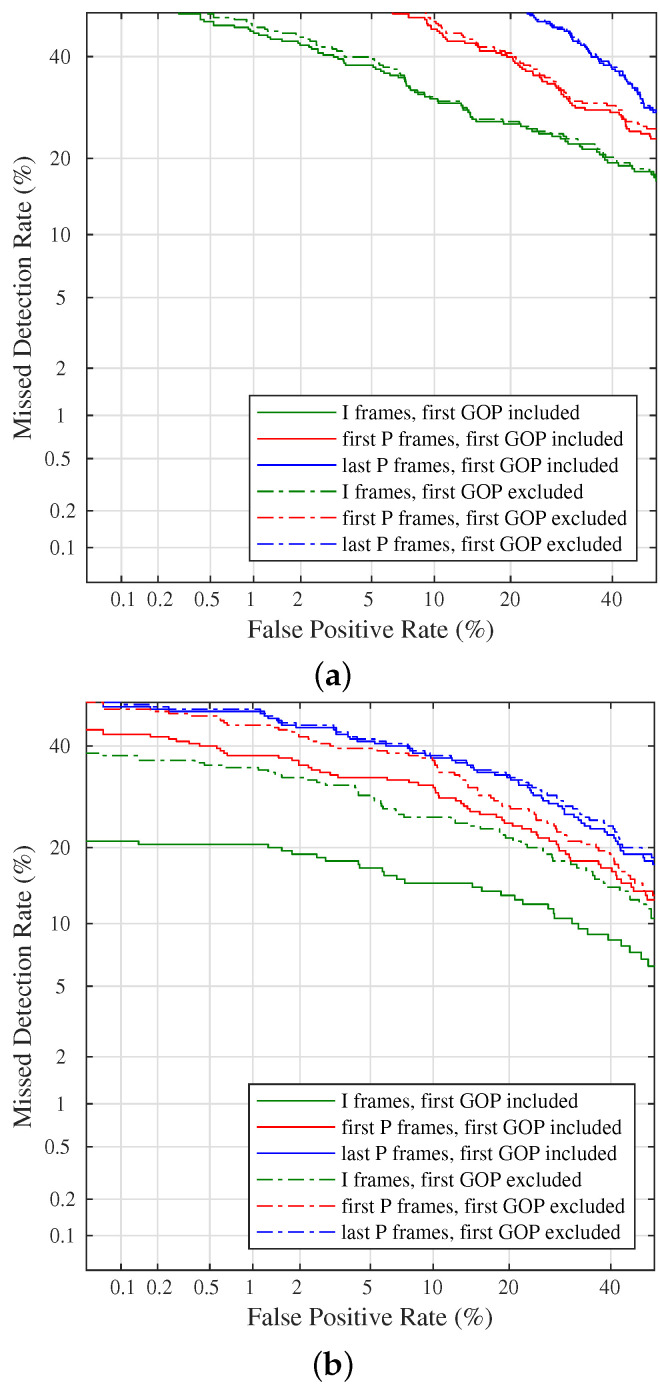
Detection Error Trade-off curves for non-stabilized (**a**) and stabilized videos (**b**). Curves are obtained by considering only I frames, only the first P frame, or only the last P frame of each GOP. The analysis is conducted by considering (solid lines) or not (dotted lines) the first GOP.

The previous analysis provides useful insights at the level of a single frame. At the video level, we applied Equation (3), and then we performed a binary classification. We describe the performance through Detection Error Trade-off (DET) curves, produced by considering only I frames or only P frames in a given position within the video stream and considering or not the first GOP. We draw them by varying the decision threshold and by calculating the False Positive Rate (i.e., the rate of true negative samples wrongly classified as positive) and the Missed Detection Rate (i.e., the rate of true positive samples that are erroneously classified as negative). Finally, the axes are non-linearly scaled by their standard normal deviates in a logarithmic scale. The DET curves are separately drawn for non-stabilized and stabilized videos (Figure 4a,b respectively). For the sake of visualization, we report results obtained using I frames, the first P frames, or the last P frames only.

A first observation rises: performance over stabilized videos seems better than non-stabilized videos over the VISION dataset, independently from the type of frame considered. This outcome is quite unexpected because the PRNU footprint of non-stabilized videos should not be misaligned, and we should have observed higher performance for non-stabilized videos. Either can explain this phenomenon by poor quality reference PRNU for some devices (particularly in devices D023 and others) or stronger compression for the oldest devices, which usually need to produce more compressed video files to address their limited storage capabilities. Nevertheless, by analyzing the two figures separately, we still notice some interesting insights. In Figure 4a we remark that I frames provide better discrimination capabilities with respect to P frames. This result is in agreement with most of the previous works, which selected I frames, only. Moreover, the position of the I frames within the entire GOP does not produce relevant differences.

Regarding stabilized videos, we remind to Figure 4b, showing that the use of I frames performs better than other strategies. However, if the first GOP is excluded, I frames and the first P frames have worst and not far discrimination capabilities (green and red dot lines in Figure 4b). Conversely, the second video frame, provides PRNU information close to most I frames (red line and green dot line, respectively). This outcome can be explained by considering how EIS works: because for some devices it is not applied to the first (I) frame, the following one (first P frame of the whole video) is weakly affected by EIS, since the content of the frames are quite correlated each other for small time intervals. Coherently, the last P frames do not contain PRNU information, independently from the considered GOP.

**Figure 5:**
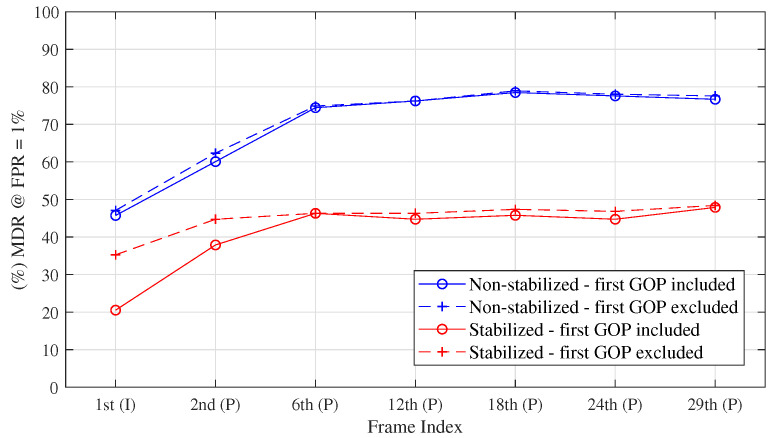
Missed Detection Rate (MDR) at 1% of False Positive Rate (FPR), obtained by considering frames at a different positions within the GOP. In blue, the results for non-stabilized videos, in red for the stabilized ones. Solid lines are obtained by considering all GOPs in a video, dot lines by excluding the first GOP.

We extended the previous analysis to frames in other positions (see Figure 5). We show the Equal Error Rate (EER) computed for the DET curves obtained by considering the frames in position {1st, 2nd, 6th, 12th, 18th, 24th, 29th}, for each GOP. It appears even clearer that for non-stabilized videos, the choice of I frames minimizes the classification error. However, P frames still contain some PRNU traces, independently from the position of the GOP. For stabilized videos, the first frame usually provides the most discriminative information, independently from the presence or absence of stabilization. Similar conclusions can be made for the first P frame, which is often slightly affected by EIS, thus providing useful PRNU traces.

**Figure 6:**
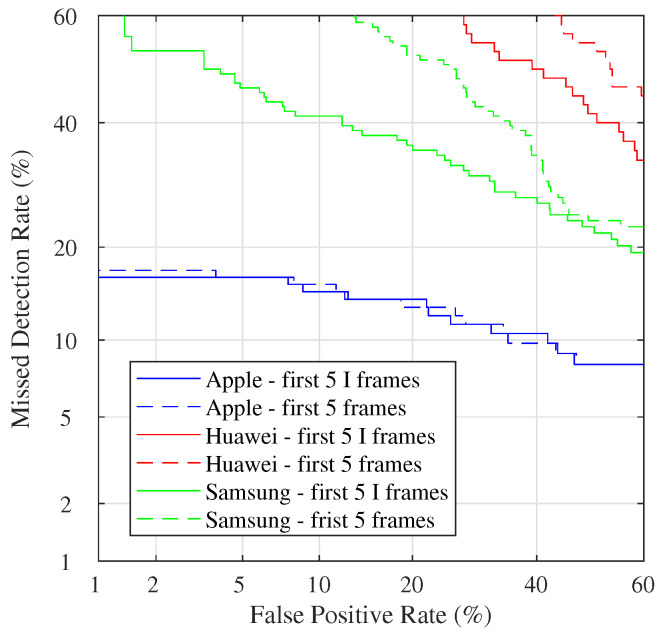
DET curves grouped for different brands available in VISION: Apple (blue), Huawei (red), and Samsung (green). Comparison of two possible strategies: taking the first five I frames (solid lines) and the first five frames of the first GOP (dotted lines).

As a the last experiment, we compared two different strategies: the first (suggested in [18]) is to use a very limited number of I frames; the second one is to consider first five video frames, independently from their type. The results are grouped by brands available in VISION (see Figure 6). It is worth to note that performance can strongly vary among different brands. Overall, the detection rate of the Apple brand is the highest. This fact is relevant if we consider that the different behaviors among brands are never considered in most proposed methods. This fact also exposes that methods’ performance can significantly change by adding or removing a few devices from a specific brand. We can fairly notice that the discrimination power is almost the same for Apple devices between the two approaches. At the same time, it is quite worse for Huawei and Samsung models (with a preference for the use of I frames only).

## 5. Findings and Insights

The above analysis comes up with some findings and insights that can be exploited to design a PRNU-based source video attribution task. In the following, we summarize them.

**Finding** **#1** a strategy that could be optimal both in presence of EIS and not is difficult to be achieved. In fact, the literature [21,31] shows that for non-stabilized videos, the optimal solution is to aggregate PRNUs extracted from all frames, or at least all the I frames, according to Equation (1). At the same time, this approach can hardly be applied to the case of stabilized videos. Conversely, working at frame level, [18] improves results for stabilized videos, but it is sub-optimal for non-stabilized videos.
**Finding** **#2** from the previous finding, we can derive that a system able to classify whether a video is stabilized or not reliably could lessen the problem. Some techniques exist, such as [20], but if used, the final accuracy is clearly affected by the uncertainty that this tool introduces. Moreover, when this solution is adopted in large-scale scenarios, the complexity that the solution introduces becomes not negligible.
**Finding** **#3** the overall performance of source attribution based on PRNU heavily depends on the device model and/or brand.
**Finding** **#4** I frames indeed convey a less attenuated and distorted PRNU independently from the presence/absence of EIS and the source brand.
**Finding** **#5** In presence of EIS, the first I frame usually provides the most significant PRNU information. Depending on the device, it can be affected or not by EIS.
**Finding** **#6** P frames could contain a weakly distorted-attenuated PRNU in both cases. In presence of EIS, we found that P frames within the first GOP generally provide the most reliable PRNU information.
**Finding** **#7** When possible, P frames that follow an I frame should be preferred. Except for this, the contribution of P frames does not depend on their position within the GOP.

By following the previous findings, we provide some possible strategies in function of the use cases.

**Insight** **#1** If a reliable system for classifying a video as stabilized or not is provided, it should be adopted in order to perform two different matching strategies (global accumulation vs. frame-level analysis). The final performance should take into consideration the accuracy of the whole system.
**Insight** **#2** If the video duration is long enough and with a sufficient number of I frames (a 1-min length video, encoded at 30 fps and with a GOP size of 30 frames, contains at least 60 I frames), the choice of using only I frames is a good trade-off between performance and complexity. The first I frames should be privileged in the analysis.
**Insight** **#3** If the video is short and the number of I frames is limited, P frames could be exploited to improve performance. In particular, the first P frames following an I frame are proved to be more suitable than the others.
**Insight** **#4** When deciding a threshold for the binary decision, a training with a control dataset should be adopted. The dataset should be as close as possible to the target devices in terms of EIS and video encoding to avoid unexpected classification errors.

## 6. Conclusions

In this paper, we evaluated which type of frame is more useful to perform PRNU-based video source attribution, even in presence of electronic image stabilization. We systematically analyzed the PRNU contribution of each video frame in order to assess if an optimum unified strategy could be performed, independently from the adoption of electronic image stabilization and the device model. The analysis shows that such a strategy still remains far from being defined, since we discovered that video compression behavior and EIS implementations can strongly vary among brands. At the same time, we provided some findings and insights that the forensic practitioner should take into account to optimize the source attribution strategy depending on the investigated device and the available information.

## Figures and Tables

**Table 1:** Frame-level analysis: second and third columns show the percentage of I frames and P frames, respectively, providing a PCE higher than a threshold Th ≥ 50. The remaining columns provide the standard deviation of the shift associated with each correlation peak whose energy is higher than the threshold. Devices adopting EIS are highlighted in bold. PRNU information can be found more easily on Apple devices and some fewer spread models (e.g., Lumia 640 LTE, Ridge 4G). Conversely, in Samsung and Huawei devices, the contribution of a single frame is rather enough to identify the source, especially for inter-coded ones correctly.

Brand	Model	Device	(%) I Frames	(%) P Frames	$\left(\sigma_{x},\sigma_{y}\right)$ on I Frames	$\left(\sigma_{x},\sigma_{y}\right)$ on P Frames
Apple	**iPhone 4s**	**D02**	**50.74**	**12.63**	**(6.16, 9.87)**	**(6.50, 8.68)**
	**iPhone 5c**	**D05**	**11.76**	**13.21**	**(3.48, 0.92)**	**(1.11, 1.06)**
	**iPhone 6**	**D06**	**35.69**	**7.97**	**(3.80, 4.45)**	**(2.95, 3.53)**
	iPhone 4	D09	85.22	51.37	(0.44, 0.00)	(0.55, 0.05)
	**iPhone 4s**	**D10**	**5.37**	**0.06**	**(6.55, 6.47)**	**(3.69, 4.21)**
	**iPhone 5c**	**D14**	**7.36**	**8.64**	**(4.72, 4.92)**	**(5.92, 9.91)**
	**iPhone 6**	**D15**	**37.73**	**23.52**	**(3.89, 4.35)**	**(2.60, 3.04)**
	**iPhone 5c**	**D18**	**25.65**	**26.07**	**(0.42, 0.61)**	**(1.19, 1.18)**
	**iPhone 6 Plus**	**D19**	**52.50**	**25.54**	**(4.88, 5.42)**	**(3.70, 4.09)**
	**iPad Mini**	**D20**	**36.48**	**19.85**	**(5.93, 7.34)**	**(3.92, 4.27)**
	**iPhone 5**	**D29**	**9.62**	**6.20**	**(2.01, 2.10)**	**(2.43, 2.07)**
	**iPhone 5**	**D34**	**10.46**	**13.20**	**(0.92, 2.94)**	**(1.08, 2.04)**
Huawei	P9	D03	1.84	0.03	(0.00, 0.38)	(0.00, 0.00)
	P9 Lite	D16	0.00	0.00	-	-
	P8	D28	2.68	0.00	(0.00, 0.23)	-
	Honor 5c	D30	0.00	0.00	-	-
	Ascend	D33	2.64	0.04	(0.00, 0.00)	(0.00, 0.00)
Samsung	Galaxy S3 Mini	D01	1.20	0.04	(0.28, 0.00)	(0.00, 0.00)
	Galaxy Tab 3	D08	0.00	0.00	-	-
	Galaxy S3	D11	18.61	1.64	(0.00, 0.00)	(0.15, 0.15)
	Galaxy Trend Plus	D22	0.97	0.00	(0.00, 0.00)	-
	Galaxy S3 Mini	D26	0.00	0.00	-	-
	Galaxy S5	D27	3.33	0.10	(0.46, 0.50)	(0.50, 0.51)
	Galaxy S4 Mini	D31	1.94	0.00	(0.00, 0.00)	-
	Galaxy Tab A	D35	18.19	0.83	(0.00, 0.46)	(0.52, 0.49)
OnePlus	**A3000**	**D25**	**17.50**	**7.84**	**(3.74, 2.69)**	**(0.79, 0.91)**
	**A3003**	**D32**	**22.50**	**17.34**	**(4.78, 4.64)**	**(2.30, 2.43)**
LG	**D290**	**D04**	**0.00**	**0.00**	**-**	**-**
Lenovo	P70A	D07	0.14	0.03	(0.00, 0.00)	(0.00, 0.00)
Sony	**Xperia Z1 Compact**	**D12**	**22.08**	**9.40**	**(0.77, 0.76)**	**(0.75, 0.69)**
Microsoft	Lumia 640 LTE	D17	33.33	12.08	(0.45, 0.00)	(0.48, 0.08)
Wiko	Ridge 4G	D21	26.51	11.77	(0.00, 0.00)	(0.04, 0.00)
Asus	**Zenfone2 Laser**	**D23**	**0.00**	**0.00**	**-**	**-**
Xiaomi	Redmi Note 3	D24	0.97	0.02	(0.53, 0.38)	(0.55, 0.45)

## Data Availability

Publicly available datasets were analyzed in this study. They can be found here: https://lesc.dinfo.unifi.it/VISION/, accessed on 12 February 2022.

## Abbreviations

The following abbreviations are used in this manuscript:

B frame	Bidirectional predicted frame
DCT	Discrete Cosine Transform
DET	Detection Error Trade-off
EER	Equal Error Rate
EIS	Electronic Image Stabilization
GOP	Group of Pictures
HVS	Human Vision System
I frame	Intra-coded frame
MB	Macro Block
MDR	Missed Detection Rate
P frame	Predicted frame
PCE	Peak-to-Correlation Energy
PRNU	Photo Response Non-Uniformity
LD	Linear dichroism
